# Towards Valorization of Food-Waste-Derived Pectin: Recent Advances on Their Characterization and Application

**DOI:** 10.3390/molecules28176390

**Published:** 2023-09-01

**Authors:** Ilaria Frosi, Anna Balduzzi, Giulia Moretto, Raffaella Colombo, Adele Papetti

**Affiliations:** 1 Department of Drug Sciences, University of Pavia, 27100 Pavia, Italy; ilaria.frosi01@universitadipavia.it (I.F.); giulia.moretto01@universitadipavia.it (G.M.); raffaella.colombo@unipv.it (R.C.); 2 Center for Colloid and Surface Science (C.S.G.I.), University of Pavia, 27100 Pavia, Italy

**Keywords:** pectin, food waste valorization, circular economy, sustainability, encapsulating agent, food packaging

## Abstract

Pectin, a natural biopolymer, can be extracted from food waste biomass, adding value to raw materials. Currently, commercial pectin is mostly extracted from citrus peels (85.5%) and apple pomace (14.0%), with a small segment from sugar beet pulp (0.5%). However, driven by high market demand (expected to reach 2.12 billion by 2030), alternative agro-industrial waste is gaining attention as potential pectin sources. This review summarizes the recent advances in characterizing pectin from both conventional and emerging food waste sources. The focus is the chemical properties that affect their applications, such as the degree of esterification, the neutral sugars’ composition, the molecular weight, the galacturonic acid content, and technological–functional properties. The review also highlights recent updates in nutraceutical and food applications, considering the potential use of pectin as an encapsulating agent for intestinal targeting, a sustainable biopolymer for food packaging, and a functional and emulsifying agent in low-calorie products. It is clear from the considered literature that further studies are needed concerning the complexity of the pectin structure extracted from emerging food waste raw materials, in order to elucidate their most suitable commercial application.

## 1. Introduction

According to annual Food and Agriculture Organization (FAO) data on food loss and waste [1], there has been an increasing interest in the reutilization of organic byproducts, as they boast a high presence of bioactive secondary metabolites, dietary fiber, essential oils, and pigments that can be recovered to produce new raw materials. 

Nowadays, there is a growing demand for pectin in the market, as it represents a sustainable biopolymer with many applications in food, pharmaceutical, and cosmetic sectors. In fact, the demand for functional food, low-fat and -calorie products, and food additives to obtain better texture, as well as for thickener and stabilizer agents and skin anti-aging, drove the growth of this market [2,3]. A recent report valuated the global pectin market at USD 1.19 billion in 2021, with a forecasted revenue of 2.12 billion in 2030, indicating a compound annual growth rate of 6.5% during the forecast period [4]. 

**Figure 1:**
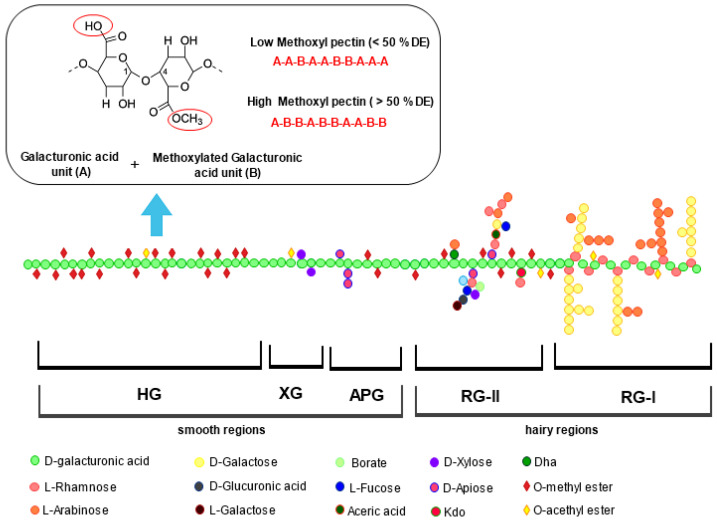
Schematic representation of pectin composed by smooth regions of homogalacturonan (HG), xylogalacturonan (XG), apiogalacturonan (APG), and hairy regions of rhamnogalacturonan-I (RG-I) and rhamnogalacturonan-II (RG-II). General structure of low and high methoxy pectin is also reported.

Pectin is a class of heteropolysaccharides located in the plant cell wall of fruit and vegetable pulps and peels [5]. It comprises approximately 65% homogalacturonan (HG) in the smooth region, which consists of a linear backbone of GalA units linked with a-(1,4)-glycosidic bond. The remaining 35% is a ramified structure containing rhamnogalacturonan (RG-I and RG-II) in the hairy region, along with xylogalacturonan (XG), and apiogalacturonan (APG) [5,6]. Depending on the food source and extraction approach used, HG can be highly or partially esterified with methanol. The esterification level and the GalA content are strictly related to the degree of methoxylation (DM) and acetylation (DA), representing the number of methoxyl and acetyl groups that can substitute the carboxylic acid groups in the GalA residues. Based on DM, also reported as the degree of esterification (DE), pectins are classified as high methoxyl pectin (HMP having DM > 50%) and low methoxyl pectin (LMP having DM < 50%) [7] (Figure 1). These parameters have a crucial impact on the commercial properties of pectin, and therefore on their industrial application. 

To meet the standards set by EU regulation No. 231/2012 for food-grade pectin, the galacturonic acid (GalA) content must be at least 65% [8]. Currently, commercial pectin (coded E440a for Low Molecular Pectin—LMP and High Molecular Pectin—HMP, and E440b for amidated pectin) is mainly extracted from citrus peels (85.5%), and apple pomace (14.0%), with a small segment from sugar beet pulp (0.5%) [9]. However, the increasing interest in the valorization of food byproducts and the high global market demand have led researchers to explore emerging sources of pectin. Several studies have focused on the extraction and characterization of pectin from different agro-industrial wastes, such as mango and banana peels [10,11], pumpkin and melon peels [12,13], cocoa pod husks [14], eggplants [15], tomatoes [16], and potato peels [17]. These alternative sources effectively captured the attention of researchers as potential sustainable alternatives for pectin production. 

In this review, the literature published in the last 7 years (2016–today) on food-waste-derived pectin was collected, with a specific focus on their extraction yield and properties that play a crucial role in defining their applications. These properties include DM and GalA content, as well as emulsifying and rheological properties, which are closely related to the sources and extraction methods used. Moreover, an overview on their recent applications in the nutraceutical and food field is presented, highlighting promising updates on their use as encapsulating agents, biopolymers for food packaging, functional foods, and emulsifying agents. By comprehensively examining the potential of food waste as a source of pectin, we contribute to the ongoing efforts in creating a circular economy and reducing the environmental impact of waste disposal.

## 2. Sources of Food-Waste-Derived Pectin

This section focuses on the main sources of pectin derived from food waste. 

A detailed overview of the type of pectins that can be extracted from conventional and emerging sources of food waste is presented, considering their extraction yield and crucial physicochemical features. 

**Figure 2:**
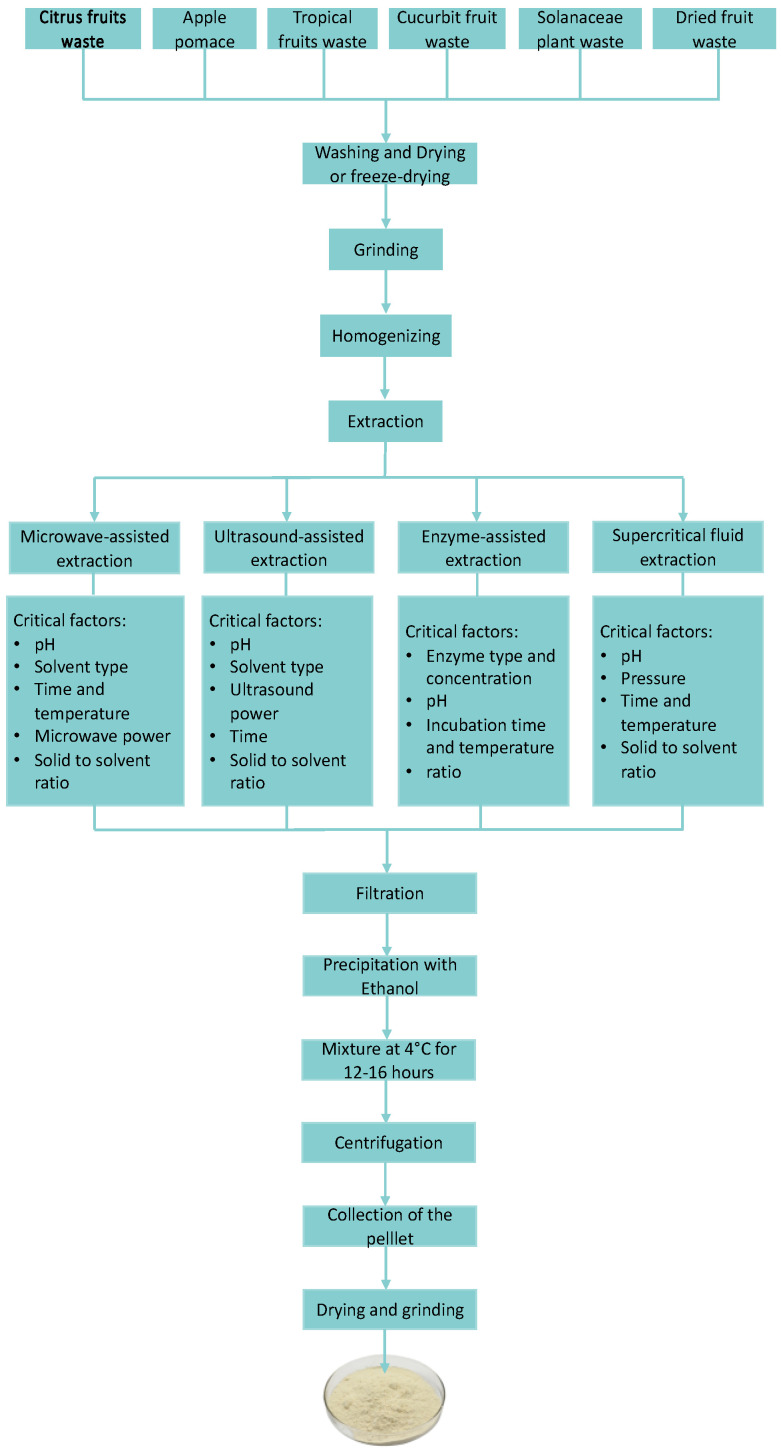
A flow diagram of pectin extraction from different food wastes.

A flow diagram of the extraction of pectins from different food wastes is presented in Figure 2. Exploring the use of different extraction methods can enhance the extraction efficiency of this polysaccharide, thereby finding new sources for industrial applications. In Table 1, a summary of the highest extraction yields of pectin obtained from the different food waste sources reviewed is reported. The data presented can guide researchers towards promising sources that could be further investigated to meet the increasing demand of the sustainable pectin biopolymer.

### 2.1. Conventional Sources

#### 2.1.1. Citrus Fruit Waste

According to FAO [35], citrus is one of the most popular and widely grown fruit crops, belonging to the *Rutaceae* family. Over the past three decades, world citrus production has constantly grown and approximately one-third of citrus fruits is used for processing, leading to the production of approximatively 50–60% of organic waste [36]. Recent advances have been registered in the valorization of citrus fruits’ processing waste, especially pectin, that can be usually obtained from orange and yellow citrus fruit types, including an orange and mandarin, lemon, grapefruit, and lime [17]. 

It is known that several researchers study the citrus pectin components by using different extraction techniques, solvents, and process parameters. Depending on these variables, the obtained yield is different. In regards to orange-colored fruit wastes, the highest pectin yield (25.6%) was obtained from a mandarin peel by Colodel et al. [18], using a conventional acidic extraction (CE) with nitric acid (pH 1.6; time, 100 min; liquid–solid ratio—LSR, 36 mL g^−1^), differently from Polanco-Lugo et al. [37] and Zhang et al. [38] who obtained lower yields, i.e., 10.06% ± 0.38 and 4.2% ± 0.7 using citric and hydrochloric acid, respectively. Differently, a very interesting innovative technique was applied by Patience et al. [39] to an orange peel. It consists of a continuous and pulsed ultrasound extraction (PUAE). Besides being a green technology, ultrasounds enable facilitating reactions with reduced energy loss, without affecting the quality of the treated matrix. For the first time, the effect of ultrasound power and pulse sonication on the extraction yield was simultaneously investigated. Seventy percent nitric acid (pH 2–3) was used as an extraction solvent and a 500 W ultrasonic horn with and without pulses at intervals of 1 s and amplitudes of 20, 40, and 60% with an associated power of 11.5, 24.3, and 34.2 W was considered, respectively. The extraction yield linearly increased with the increasing amplitude/power and the pulse mode was the winning strategy for the next step of the scale-up process, as it generated less energy than continuous irradiation, but it increased the availability of power and energy storage capacity. However, this process resulted in a lower yield (11%) than that obtained by combining the surfactant and microwave-assisted extraction (s-MAE). In fact, after an optimization of MAE conditions through a Box–Behnken design and the addition of Tween 80 (pH 1.2, LSR 1:21.5 (*v*/*w*), 7 min, 400 W, and surfactant Tween 80 8 g L^−1^), commercially available food-grade HMP was obtained from an orange peel with a yield of 32.8 ± 0.8%. In addition, this pectin was characterized by 69.8 ± 0.4 DM, 78.1 ± 2.0% GalA, and 286.3 ± 5.2 kDa MW [19]. 

Recently, the research has been focused on the combination of green techniques such as MAE, ultrasound-assisted extraction (UAE), and enzyme-assisted extraction (EAE), which give a higher yield in a shorter time, aiding the extraction of a high-quality pectin. Moreover, according to the green chemistry principles, mineral acids can be replaced by organic acids, such as acetic and citric acids, also showing a higher hydrolyzing ability [9]. According to a study of Sayah et al. [20], the pectin type varies according to the citrus species waste used as raw material, and to the nature of the acid used, which significantly affects pectin yield and MW. In particular, the extraction yield for both orange and grapefruit peels increased when a residual peel obtained after essential oil removal with hydrodistillation was used as raw material instead of a fresh peel, because of the weakened peel structure obtained, which enables a stronger interaction between the acidic solvent and the matrix, independently from the acid used. Conversely, the MW of pectin extracted with sulfuric acid was lower than that of pectin extracted with citric acid. In addition, citric acid caused a greater increase in the intrinsic viscosity of the extract than the sulfuric acid, showing a positive effect on DM.

Concerning yellow-colored citrus fruits, pectin yields obtained from a peel reported in the literature are in line with those obtained using the orange ones, varying between 16.12% and 28.57% [20,21,40,41]. Moreover, lime and lemon peels could be considered a promising source of highly esterified pectins, showing good emulsifying properties and a potential application in the food industry as food additives [41].

Considering the rheological properties of citrus pectin, considerable scientific research has proven that it has excellent gelling, thickening, and water holding capacities, in addition to an encapsulating property, which provide its optimal adaptability to industrial fields like food processing and packaging, nutraceuticals, drugs, cosmetics, and personal care products [17]. Despite this, depending on the citrus fruit or for the same fruit on the different waste, pectin differs in its structural configuration, carbohydrate, and methyl ester group distribution. Consequently, different structures can modify pectin emulsifying and rheological properties, as demonstrated by Dimopoulu et al. [42]. Specifically, in this study, a pectin isolation protocol from different parts of lemon fruit, including core parts and membranes (LCP), juice (LE), and albedo (LA), was reported. After a dialysis step, which was used to remove contaminants, the yield obtained with hot-acid extraction (pH range 1.5–3.0) was the highest for LA (80 g kg^−1^), followed by LCP (45 g kg^−1^) and LE (10 g L^−1^). The average MW ranged between 75 × 10^3^ g mol^−1^ and 193 × 10^3^ g mol^−1^, thus indicating HMW-type pectins, mainly composed of GalA with a low protein content, differing in sugar composition and the degree of methylation depending on pectin backbone variations; in fact, LA pectin was more methylated than those from LCP and LE. Moreover, LA pectin (at 20 g L^−1^) had the lowest viscosity, whereas intrinsic viscosity indicated that LCP and LE pectin had a greater hydrodynamic volume than LA pectin, in agreement with literature data and MW measurements. Nevertheless, all isolated lemon pectins increased the viscosity by increasing their concentration, showing a pseudoplastic behavior, useful for pharmaceutical liquid formulations and for a potential use in food and drink products as a suspending agent and viscosity enhancer. Comparing citrus peel pectin with other conventional vegetable pectin sources (for example, cereals and soybeans), it was evident that it had a higher water solubility, water holding capacity (WHC), and viscosity, and therefore it formed a strong bond with water present in the food matrix, thus reducing syneresis during food storage [43]. Based on this aspect, “hot-water”-based extraction methods are more efficient and enable obtaining high-quality pectin, especially in the presence of citric acid, leading to the highest extraction yield and DM% (21 and 82.5%, respectively) [44]. 

Another important characteristic of citrus pectin is the effect of the mesoscopic structure of citrus pectin underlining the importance of its compactness on the emulsifying properties. The pectin mesoscopic structure represents a three-dimensional conformation with a variable extent ranging from few tens of nanometers to micrometers, generated by intra- and inter-molecular forces among the functional groups, after hydration; the acidic pH and ionic strength of the solution can modulate not only pectin size and compactness but also mesoscopic structures. In fact, a less mesoscopic structure, but more compact, is obtained for pH- and NaCl-regulated pectin because it generates a compact absorbed layer at the emulsion interface able to resist droplet coalescence/flocculation during homogenization, revealing that compactness is more important than size. Therefore, this could represent a strategy to improve the emulsifying ability of citrus pectin, which is more methyl-esterified than other pectin sources [45].

#### 2.1.2. Apple Pomace

As mentioned above, besides citrus fruits, apple waste is one of the most abundant and promising sources exploited for pectin extraction and characterization [46]. The extraction yield of apple pectin ranges from 6.4 to 22%, depending on extraction parameters (pH, temperature, heating time, sample, and LSR). Pectin extraction from discharged apple pomace using food-grade organic acids (tartaric, malic, citric, and generally recognized as safe—GRAS) could represent an eco-friendly and safe protocol leading to a yield (6–7%) similar to that obtained using 0.1 M HCl, widely used in the pectin manufacturing industry with additional toxic chemical waste production. Moreover, the extraction process with citric acid resulted in high-viscosity apple peel pectin [22,47]. Nevertheless, a good apple pomace pectin yield (about 10%) was also obtained using MAE and radiofrequency-assisted extraction (RFAE) [23], differently from what was obtained using CE, probably due to the prolonged time and high temperature applied, which caused the de-esterification of polygalacturonic chains. In particular, RFAE is suitable for larger site material heating, thanks to the uniformity of the electromagnetic field and to the great penetration depth [23,48]. A high extraction yield (14.89%) also derives from a subcritical water extraction (SWE) process [24], confirming that innovative extraction techniques are more efficient than the traditional ones. Concerning SWE, temperature has a higher effect on pectin extraction than the extraction time; in fact, low (100–120 °C) and high (140–160 °C) temperatures lead to HMW and LMW pectin, respectively; LMW pectin, in particular, is interesting because when digested, it promotes the growth of microbiota and therefore it could be suitable for the development of functional foods. 

Also for apple pomace pectin extraction, the main advantages deriving with the use of innovative techniques instead of the traditional methods are mainly higher yields; less time and time/energy consumption, especially for MAE, which is also less solvent-consuming, thus avoiding matrix depolymerization problems; and finally low equipment corrosion [46]. This pectin, which represents 14% of commercial pectin, is characterized by HMP with a DM of 50%, pointing out its suitability as a potential gelling agent; in addition, it also fits the quality standard of commercially available food-grade pectin (GA 65 g/100 g) recommended by the Joint Expert Committee on Food Additives (JECFA) [22,49].

### 2.2. Emerging Sources

#### 2.2.1. Tropical Fruit Waste (Banana, Mango, Papaya, Passion Fruit, and Jackfruit)

In recent years, tropical-fruit-derived waste such as mango [13,25,50], jackfruit [27,51], banana [14,26], papaya [52], custard apple [53], and passion fruit peels [54] have been proposed as interesting sources of pectin. As already described for citrus byproduct pectin (Section 2.1.1), the use of innovative and sustainable hybrid extraction techniques was encouraged. For example, Zhao et al. [54] combined the cavitation effect of an ultrasonic wave and the electromagnetic heating of microwave treatments on a fresh passion fruit peel, leading to an increase in polysaccharide yield and an enhancement of its thermal stability, steady-state fluid property, and viscoelastic behavior. Considering that the extraction method significantly affects pectin MW, Li et al. [27] compared an eco-friendly subcritical water method with a citric-acid-based one for the extraction of jackfruit (*Artocarpus heterophyllus* Lam.) peel pectin. MW was significantly higher (174.3 kDa) when pectin was extracted using the citric-acid-based method and characterized by more hairy regions and side chains. Consequently, the gel strength, stickiness, viscous force, and hardness (considered as the gel property evaluation index) were higher, considering the direct correlation of MW with gelling properties. Differently, no significant difference was registered for GalA content and DM was in both cases higher than 50%. A correlation between jackfruit waste pectin (JWP) structural characteristics and its textural properties was also confirmed by Begum et al. [51]. In fact, JWP formed gels, even with lower gel strength than analytical-grade pectin and commercial pectin. However, a harder gel strength could be generated using ammonium oxalate instead of sulfuric acid to extract JWP. Concerning JWP use, it could mainly be used as a gelling agent in marmalades, jams, and fruit suspensions, especially when a low sucrose concentration is present, as sucrose concentration higher than 55% led to localized pectin aggregation, thus compromising the homogeneity of the three-dimensional hydrogen-bonded structure formed by pectin in water [51].

Factors affecting the physicochemical properties of pectin extracted from tropical and subtropical fruit byproducts and their application include the origin of the plant material and the ripeness degree of the fruit [9], as evident from a chemometric analysis showing significant differences among four mango varieties (mahachanok, chok anan, nam dok mai, and kaew mangoes) according to their physiological attributes and peel composition [13]. This reflects the pectin recovery and type (DM value). Concerning the chemical characteristics, two classes of pectin showing different equivalent weights were obtained: the highest one deriving from ‘mahachanok’, ‘chok anan’, and ‘kaew’ varieties (1000–2000 mg mol^−1^) and the lowest one from the ‘nam dok mai’ peel (600 mg mol^−1^). Overall, these pectins had equivalent weight similar to that of citrus pectin (635.63–2219.39 mg mol^−1^) [41]. The decrease in pectin MW due to a partial degradation of the pectin side chain caused by pectolytic enzymes (polygalacturonase, pectin methylesterase, and galactosidase) was correlated to a higher fruit maturity stage of the fruit, which led to the loss of neutral sugars. Moreover, DM ranged between 56.88% and 92.93%, indicating that all mango peel pectin was the HMP type, except ‘chok anan’ pectin, which could be classified as LMP and therefore useful as a supplement in a low-sugar diet. In regards to the ripening stage, it is also correlated to pectin extractability from cells, as the ripening process provides an increase in the content of pectin loosely bound to the cell wall, which occurs in parallel with a decrease in covalently bound pectin [50], and a greater pectin content in fruit peels [25].

Considering pectin extracted from a banana peel, the thickening properties and their effects on the viscosity of a whey-protein isolate (WPI) were investigated [11], after MAE-extraction condition optimization (195 °C, LSR 8%, pH 3). The pectin purification led to a lower yield (5%), but to a higher quality (36% purity grade). The pectin–protein complex showed a shear-thinning behavior, with an improved viscosity respective to a control consisting of a WPI solution without pectin. Moreover, the in vitro digestion of orange juice fortified with this pectin showed an improvement in viscosity at a low physiological shear rate, indicating the real possibility to use it as a potential thickener for food applications [14]. Conversely, a higher extraction yield (20–24%) was obtained using 0.5 N HCl, after the optimization of temperature, time, and pH (90 °C, pH 2.5, 2.5 h) [26]. Other than a waste valorization, pectin extraction from a banana peel might be an alternative to the common pectin import and a future direction is heading towards the development of edible film/coatings from banana peel pectin.

Summing up, depending on the physiochemical characteristics, the variety, and the extraction technique used, the functionality of tropical fruit peel pectin changes [10,51,52]. The common high methoxyl groups’ content of this pectin generally limits its use as a food additive, but a packaging or pharmaceutical-drug carrier application is also possible thanks to a change in its structure through a de-esterification with alkaline treatment. Current research is going on towards the use of this potential biopolymer as a functional prebiotic ingredient [50].

#### 2.2.2. Cucurbit Fruit Waste

Cucurbitaceae is a large family of plants, also known as cucurbits, including 130 genera and 800 species, widely cultivated. More than 300 plant species are used by humans, but only 150 are expansively cultivated, and 30 are crucial for the global food production, during which a lot of fruits and vegetables are intended for disposal [55]. The most common consumed and cultivated cucurbits are watermelons, melons, and pumpkins, whose wastes have a great potential use as a pectin source. 

The main waste of a watermelon is the rind, which represents approximately 30% of the whole fruit biomass and consists of 13% (*w*/*w*) pectin, 10% (*w*/*w*) lignin, 23% (*w*/*w*) hemicellulose, and 20% (*w*/*w*) cellulose [56]. Also for this waste, the acidic extraction using a conventional heating process or innovative methods is the common procedure applied [57,58] and especially the use of citric acid during a conventional heating extraction led to the highest pectin yield characterized by methoxyl and anhydrouronic acid content (pH 2.0, 62.31 min—longer-time extraction increased DM, and LSR 35.07 mL g^−1^). In fact, a suitable acidic environment favored the loss of charged carboxyl groups, the reduction in repulsive charges, and the methoxylation, thus promoting the precipitation and, consequently, the yield increasing [28]. Considering innovative extraction approaches, the best pectin properties in terms of DE, DM values, and GalA content were obtained using MAE with acetic acid and the extraction yield significantly increased with the rising of heating power and irradiation time (best conditions: 279.3 W microwave power and 12 min of irradiation time, with a yield of about 6% dry weight) [59], due to a higher solvent penetration into the material and to a rapid transfer of energy towards the solvent and the material, generating a dissolution of extracted pectin in a short time. Due to its high DE, watermelon rind pectin could be suitable to stabilize an emulsion rich in oil, because the presence of hydrophobic groups makes the polysaccharide dispose at the interface between oil and water [60]. Thus, a watermelon rind is a potential source of pectin with emulsifying properties, which can be used for food and pharmaceutical applications. In addition, watermelon rind pectin aqueous solutions (5%, *w*/*w*) can be used as thickening agents due to their relatively high viscosity [61]. 

Concerning the potential use of unutilized pumpkin biomass as a source of pectin, Baissise et al. efficiently extracted it from a peel and pulp using HCl (pH 1.8), at 80 °C, for 60 min. The pectin yield was higher for the peel (55.14 ± 0.10 g/100 g) than pulp (52.91 ± 0.02 g/100 g) and GalA content was lower than 65% with a DM of 63.88 ± 0.12%; therefore, the pectin can be classified as HMP [62]. Considering the effect of different acids on extraction yield and pectin properties, a slightly higher extraction capacity of nitric acid than citric acid, although the difference was not significant, was registered when the extraction was performed with Soxhlet (which provided a higher yield) or using conventional heating with a shaking water bath. Both methods led to pectin with a high DE (66%) with low methoxyl (6%) and acetyl content (0.43%) [63]. MAE again provided pectin with higher purity (GalA, 73.8%), even with an extraction yield similar to that obtained with the previously mentioned conventional extraction methods (about 7%) [64]. Considering the above-mentioned, pumpkin byproducts could be considered a promising alternative source for pectin material, as also recently stated by Lalnunthari et al., who isolated pectin from seeds and peels with about 73% of yield and used it to develop edible films. The data obtained from the analysis of mechanical and barrier properties were within the acceptable range and therefore this pectin can be successfully used as film for food application [29]. Moreover, pumpkin pectin (1% *w*/*w*) possessed strong emulsifying activity and could be used to substitute Arabic gum (15%, *w*/*w*), soybean soluble polysaccharides, or other types of emulsifiers used at concentrations greater than 1% [65].

Recently, MAE was successfully used to isolate pectin from a cantaloupe peel and rind, with a good extraction yield (32.81% and 18.14%, respectively). In this case, by increasing irradiation power, an increase in the yield was registered; conversely, by increasing extraction time over 12 min and 112 s for the peel and rind, respectively, the decomposition, degradation, and hydrolysis of the polysaccharide occurred [30,66]. 

Concerning the above, a good yield obtained using an optimized conventional heating citric-acid-based process and MAE has also been reported for melon peel extraction. A GalA content ranging from 40 to 48% (therefore, pectin can be classified as LMP) and a DM ranging from 19 to 29% (the high variability was due to the increase in the de-esterification occurring when the long time, low pH, and high temperature were applied) were obtained for the acidic extraction [67]; differently, a DM higher than 50% was obtained with MAE extraction (112 s) [66] and pectin was characterized by good emulsifying properties for concentrations in the range 3.0–5.0%, excellent gelling potentials, and a thickening property at higher concentrations, which could improve the foaming stability and textural strength in final pectin-based products [30]. Finally, the stability when used as an emulsifying agent is higher at a low temperature (4 °C) for both a very short period (after 1 day) or longer period (30 days) [67].

#### 2.2.3. Solanaceae Plant Waste

Within the Solanaceae family, tomatoes, potatoes, and eggplants are the most widely consumed and industrially processed vegetables, whose wastes can be enhanced as a pectin source [31,68]. 

Different innovative and traditional methods have been developed to improve the extraction efficiency and pectin quality from tomato waste, consisting of seed, skin, and residual pulp [68]. Among the innovative techniques, UAE, MAE, and ultrasound-microwave-assisted extraction (UAME) were generally applied to treat tomato waste powder and the best conditions involved the use of citric acid under pH 1.0–1.5 and LSR 1:20 (*w*/*v*), and the UAE and MAE combination resulted in an increase in the yield [32]. This can be explained by the different extraction mechanisms underlying the two methods. In fact, the UAE cavitation effect results in cell destruction and larger pore formation on the cellular matrix surface, allowing the microwave to easily transfer the compounds to the extraction medium [32].

Today, the use of conventional acidic extraction is less common. However, the use of an acidic (HCl) medium increased the pectin extraction yield from tomato skin, seeds, and residual pulp in comparison to the use of only water, and pectin was HMP, characterized by a high GalA content (about 800 g kg^−1^) and DM of about 50%. The isolated pectin had a viscoelastic and gelling behavior like that of the commercial pectin, making it suitable for food formulations, thus enhancing the value of tomato waste in food processing [69]. 

A peculiarity of the pectin obtained from tomato waste is the color, which is an important parameter affecting the gel appearance or the possible use as a food additive. Several studies confirmed an intense coloration (red/yellow) due to the pectin ability to trap lycopene or other polyphenols, which highly depends on the extraction method and the raw material used. For example, the lycopene content was lower using citric-acid-based extraction [70], as evident from the CIEL*a*b* parameters showing low a* (a = greenness, +a = redness), high b* (b = blueness, +b = yellowness), and high L* (lightness) values. When UAME extraction was applied, pectin showed a higher L* value, indicating a lower lycopene content entrapped in pectin than for UAE and MAE extraction [71]. Probably, ultrasound pretreatment extracted lycopene but the following application of microwave heating caused its destruction and fragmentation into acetone, glyoxal, laevulinic aldehyde, and methyl-heptenone.

The peel and calyx are the main eggplant byproducts, and both are considered a good source of dietary fiber, including pectin [72], which has been effectively extracted with innovative methods, such as MAE and UAE with a higher yield for the peel. In addition, calyx GalA content was under the value stated by FAO, differently from the peel (67.4%), even if both can be classified as HMP since their DM value was higher than 50% (68.18% and 60.74% for the peel and calyx, respectively) [73]. Nevertheless, an increase in yield up to 33.64% has been observed for the peel when extracted using UAE under optimal conditions (a power of 50 W, 30 min irradiation time, and pH 1.5) [31]. It was noticed, for eggplant wastes, that ultrasonic power had a negative effect on extraction yield. In fact, an increase from 50 W to 150 W resulted in a pectin degradation and fragmentation into oligosaccharides, probably as a result of the explosion of cavitation bubbles generating a high force in the medium. Conversely, an increase in irradiation time and acidity promoted the pectin release because of the increased plant material swelling, solvent penetration into the plant cell, and cell wall destruction. Peel-derived pectin had better water and oil holding capacity (6.02 g g^−1^ and 2.60 g g^−1^, respectively), and emulsifying properties than the calyx-derived one. Therefore, it can be easily used to improve textural properties and avoid syneresis problems, and as good stabilizers in fatty food products and emulsions. 

Pectin extraction from potato peels or pulp has been carried out with organic or mineral acids, as described by several authors, and the use of citric acid at 90 °C, 90 min of extraction, and pH 2.5 lead to higher yields than the use of tartaric, lactic, nitric, phosphoric, or sulfuric acid in the same experimental conditions [74]. In fact, citric acid can effectively bind pectin due to its chelating properties, which are stronger than those registered for the other organic acids, in agreement with the results of Yang et al. [75]. For mineral acids, the extraction yields ranged from 8.38% to 9.83% with no significant differences. Conversely, pectin with a higher DM (37.45%) and DA (15.38%) content and a high proportion of the RG-I domain (>60%) has been obtained with acetic acid, probably due to a weaker hydrolysis capacity [75]. However, potato waste pectin can be generally classified as LMP with a high DA, which can play a positive role in the emulsifying process. In fact, potato pectin had an emulsion activity of 44.97–47.71% and an emulsion stability between 36.54 and 46.00%, which is related to the capacity of acetyl groups as well as the branching extent of the RG-I domain to decrease surface tension and to prevent the oil particles from aggregation. Nevertheless, the pectin obtained from potatoes has a lower GalA content (<65%) than the commercial pectin, due to the presence of starch, which can be precipitated together with pectin using ethanol treatment because it is partially hydrolyzed when extracted. Thus, the preliminary treatment of raw materials with α-amylase could lead to an increase in pectin purity [76].

#### 2.2.4. Dried Fruit Waste 

Dried fruit byproducts, such as a pistachio hull and almond or walnut and cacao husk, are other pectin alternative sources. In regards to the pistachio green hull (PGH), several authors optimized the pectin extraction process [33,77,78]. The use of MAE with an aqueous acidic solution improved the extraction yield, but reduced GalA content by increasing time and power, probably due to the de-esterification of GalA chains. The optimal operating conditions, achieved at pH 1.5, an irradiation time of 165 s, and a microwave power of 700 W (18.13% yield) led to low DM (12.1 ± 2.72%) and high GalA content (about 66.0%) pectin [77]. Lower and higher yields were obtained with UAE [78] and with conventional heating extraction using citric acid (pH 0.5, 90 °C, 30 min, LSR 50 *v*/*w*), respectively [33]. Overall, pectin from PGH had good degree of purity (GalA > 65%), low DM (<50%), good water holding and oil capacity (4.11 ± 0.34 g g^−1^ and 2.02 ± 0.19 g g^−1^, respectively) [77], and good emulsifying and stabilizing properties. 

A higher extraction efficiency for walnut green husk pectin was obtained using MAE compared to UAE even if GalA content (69%), DM (54–59%), and functional features did not significantly differ [79,80]. Differently to traditional extraction, in both UAE and MAE methods, the yield increased for low LSR extraction, as the cavitation intensity imposed on the plant tissue increased, favoring fragmentation, and preventing the electromagnetic energy from being absorbed by the exceeding solvent, leading to a decrease in energy uptake by the plant material. Indeed, the functional analysis showed a good emulsifying capacity (54–58%) and a greater emulsion stability at the lowest temperatures (4 °C) after 1 and 31 days. 

Concerning the cacao pod husk, the enzymatic extraction with Celluclast^®^ (Novozyme, Bagsvaerd, Denmark) represented an appropriate alternative to acid hydrolysis and assisted sonication with a higher yield (up to 10.20 g/100 g) but lower GalA content probably because enzymatic extraction also increased the solubilization of other compounds. However, it is an environmentally friendly alternative to chemical hydrolysis. In addition, this pectin showed non-Newtonian flow and a pseudo-plastic behavior typical of this type of biomaterial and gelling properties like those of HMP, even if it was classified as LMP according to its degree of esterification [81]. In fact, it could form gel at a low pH in the presence of sucrose, as also reported by Vriesmann et al. [82]. 

Finally, Najari et al. isolated LMP rich in GalA from an almond hull with an average yield of 26.32% *w*/*w*, which increased by increasing time, temperature, and acid pH, but decreased by increasing LSR [34]. 

### 2.3. Differences among Pectins Obtained from Each Different Food Waste Source

**Figure 3:**
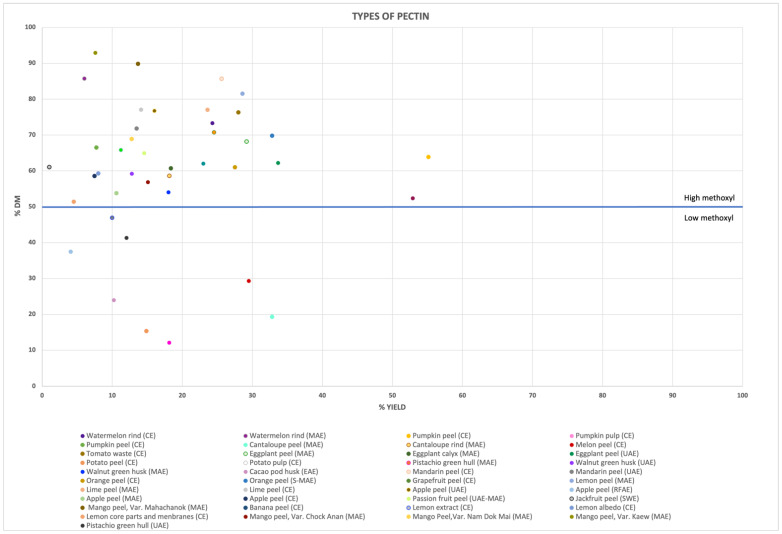
A graphical representation of the different types of pectins extracted (expressed as LMP and HMP) from various food wastes is illustrated, along with their corresponding degree of efficiency (expressed as the extraction yield). Each data point represents pectin derived from a specific food waste source, with a distinct extraction method.

Several extraction techniques have been successfully used to extract pectin with high methoxyl content from citrus and apple fruit wastes, and from tropical fruit byproducts, as illustrated in Figure 3. However, pectins extracted from sources belonging to the same botanical family do not consistently exhibit the same characteristics. Effectively, pectin extracted from a pistachio green hull using MAE and UAE methods resulted in LMP content [33,77], similar to the pectin extracted with EAE from a cocoa pod husk [81]. Conversely, pectins from a walnut green husk using MAE [79] and UAE [80] can be classified as HMP, despite the food waste source belonging to the same botanical family as previously mentioned. 

The variation in methoxyl content is also observed in Solanaceae-plant-derived pectins. In fact, tomato waste [68], eggplant peels [72], and calyx [73] provided HMP, while LMP was recovered from potato peels and pulp byproducts [74]. Similarly, cucurbit fruits generally contain HMP, with the exception of cantaloupe melon peels [66,67].

The findings deriving from the literature reviewed in this manuscript suggest that the type and methoxyl content of pectins extracted from different waste can vary significantly, even within the same botanical family. Moreover, the extraction methods and their related critical parameters significantly affect the extraction yield. However, the most crucial factor determining the type of pectin extracted is the origin of the waste material. These findings are important for better understanding the potential industrial applications of pectin deriving from various food waste.

## 3. Application in Nutraceutical and Food Field

**Figure 4:**
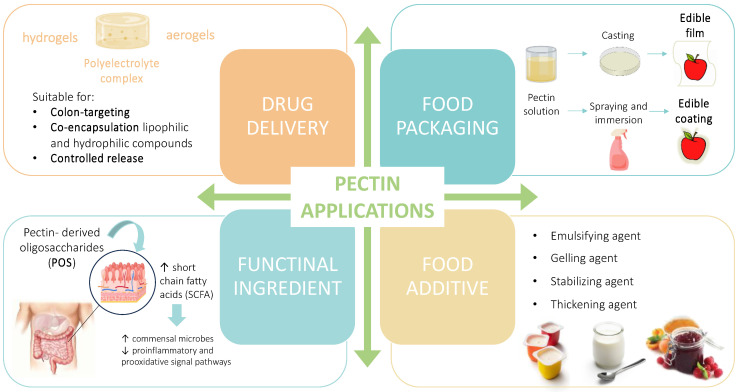
Graphical summary of the recent application of food-waste-derived pectins (↑ increase; ↓ decrease).

As it has been discussed, pectin is a highly heterogenous biopolymer and its use is receiving more and more attention. Very recent updates on the application of food-waste-derived pectin are reported in the section below (Figure 4) and summarized in Table 2.

### 3.1. Encapsulating Agent

Pectin has been extensively studied for its encapsulating ability, as it is a food-grade and biodegradable polymer that has the great advantage to be resistant to a gastric environment and to enzymatic hydrolysis in the human small intestine [95]. For these reasons, it is an optimum candidate for developing intestinal delivery systems for polyphenols, essential oils, and probiotics, increasing their solubility and bioavailability in the gastrointestinal environment [96,97,98]. 

A recent application of pectin is the development of carriers for colon targeting. Several studies reported the ability of pectin to interact well with other materials that can be added in such formulation [99]. In fact, the natural polysaccharide alone is soluble in gastrointestinal fluids and tends to swell in contact with them, resulting in an early active release before the colon. Thus, delivery systems are generally designed exploiting the ability of pectin to interact with cations, leading to the formation of hydrogels, or with polysaccharides and proteins, generating polyelectrolyte complexes [96]. LMP is generally preferred to HMP as an encapsulating agent, due to the presence of a high amount of carboxyl groups non-methyl-esterified on GalA chains that can create a rigid matrix structure by cross-linking with divalent cations, such as Ca^2+^ and Zn^2+^, and thus forming Ca-pectinate or Zn-pectinate hydrogels through an ionic gelation mechanism [99]. 

Therefore, ionic bonds between cations and GalA carboxyl groups located on two different LMP chains can be formed, which lead to the generation of an “egg box” gel structure stable in a wide pH range (from 2 to 6) [15,95]. Additional crosslinkers can be added to reinforce the formulation. For example, Lee at al. produced hydrogel beads with the ionic gelation mechanism to deliver quercetin using de-esterified pectin at different concentrations (1, 1.5, and 2%) from a yuzu (*Citrus junos*) peel (DEYPP) with Ca^2+^ cross-linking and the addition of oligochitosan. In vitro release studies showed that the quercetin release was lower than 1% for all the oligochitosan-DEYPP hydrogels in simulated gastric (SGF) and intestinal (SIF) fluids. When the beads were exposed for 12 h to the simulated colon fluid (SCF), quercetin release increased (99.54% from 1% beads, 98.72% from 1.5% beads, and 65.37% from 2% beads). Moreover, the degree of biopolymer hydrolysis and the active release were affected by DEYPP concentration with 2% DEYPP beads having a too high particle size to be sufficiently hydrolyzed by colonic enzymes [83].

In regards to the use of HMP, it could be de-esterified by applying an enzymatic treatment (i.e., using commercial pectin methylesterase) before using it for binding [83,85]. 

Pectin can also be used to produce polyelectrolyte complexes through polysaccharide–protein/peptide linking. In this case, two opposite charged polymers, such as pectin carboxyl groups and chitosan [83] or protein [96] amino groups, interact, forming a complex able to encapsulate active substances. The use of polyelectrolyte complexes could be an effective approach to co-encapsulate hydrophilic and hydrophobic bioactives. A clear example of this application is represented by pectin-coated zein nanoparticles for the delivery of tannic acid and resveratrol (a hydrophilic and hydrophobic active, respectively), showing relatively high encapsulation efficiency (about 51.5% and 77.2% for tannic acid and for resveratrol, respectively). About 59.4% of tannic acid was released in SGF after 120 min of digestion (in contrast to a total release of the free active) and a complete release was reached in the small intestinal phase within 30 min. However, these values were two-fold higher when only tannic acid was encapsulated in pectin zein-nanoparticles, highlighting that the co-encapsulation negatively affected its release. Considering resveratrol, it was released during the gastric digestion for about 42.4% (four-fold higher than the non-encapsulated) and for about 77.6% at the end of the small intestinal phase. Comparing these results with previous studies, the co-encapsulation seemed not to impact the stilbene release. However, the formulation was not effective for colon targeting, due to the total release of the bioactive at the small intestinal level [86]. Conversely, Shishir et al. developed a nanoliposome coated with a polyelectrolyte complex formed with citrus peel pectin and chitosan to deliver neohesperedin with the layer-by-layer method. The delivery system was effective to protect the flavonoid from digestion, preserving around 72% and 78% of neohesperidin during gastric and intestinal phases, respectively, thus making it suitable for colon targeting. In fact, chitosan protected pectin under gastric conditions, preventing the bioactive release and the subsequent pectin degradation by colon enzymes promoting its release [84].

Biopolymer-based aerogels have also gained increasing attention as a smart delivery for therapeutic agents with controlled release [100]. An example is represented by aerogel beads to delivery vanillin using watermelon rind pectin, which was tested as a carrier at two different concentrations. The developed beads had a high surface area and low-bulk density, making them suitable for delivery applications. Vanillin was loaded using impregnation and its release profile was affected by the affinity with pectin, which was low and in turn provided a fast release [85]. To elucidate the possible mechanism of food-waste-derived pectin aerogel under a simulated gastro-intestinal condition, the delivery of theophylline using commercial citrus pectin was investigated. The methylxanthine release was tested after 1 h in SGF (first phase) and then in SIF (second phase). In the first phase, the flavonoid resulted in a fast release governed by matrix dissolution at a low pH; in contrast, under SIF conditions, theophylline release decreased due to the matrix network created with calcium cross-linking that counteracted matrix erosion. This study highlighted the need to adjust calcium concentration and initial pectin solution pH to obtain aerogel with controlled release [101].

### 3.2. Film and Coating Agent in Food Packaging 

Pectin is also used in biodegradable food packaging and edible coatings for food preservation to improve the storage stability of food products, due to its excellent film forming ability and low toxicity [91]. It is widely studied as a sustainable alternative to replace common petroleum-based materials that generally contain harmful contaminants, such as bisphenol and phthalates [89,102]. Therefore, in this context, the most important pectin characteristics to be considered are mechanical features, humidity, and oxygen barrier properties, which are strictly related to pectin sources and to its physicochemical composition. In particular, the mechanical strength and stiffness of pectin films are correlated with GalA content, DM, and DA, which also affect the moisture barrier performance; GalA content and DM are also responsible for the oxygen barrier performance [87], as recently highlighted by a study on the characterization of fig-stalk-derived pectin films over currently commercial films. Pure stalk pectin (PSP) had comparable and higher mechanical strength than citrus and commercial apple pectin, respectively. Moreover, PSP CaCl_2_ cross-linked and not cross-linked films had the highest moisture barrier effect and therefore PSP could be an alternative hydrocolloid source with higher edible film properties than commercial pectin [87].

The combination of pectin with polysaccharides [103], proteins [104], lipids, or plasticizers, as glycerol or sorbitol [88], forms composite films, which can increase the strength, flexibility, and thickness of the coating. For example, the use of glycerol increased the workability, flexibility, and thickness of an innovative coating film based on pectin extracted from *Hibiscus sabdariffa* L. (HsL) byproducts to preserve strawberries. A central composite design was used to optimize the preparation of the coating, investigating the influence of different parameters, such as pectin (0.8–1.8%) and plasticizer (glycerol, 0.5–1.5%) concentration, and casting volume (10–20 mL). Pectin concentration strongly affected the viscosity of the coating solutions, due to the HMP content, which is responsible for the material gelling capacity. The obtained optimal coating solution (1.6% of pectin and 1.3% of glycerol) fit well with the parameters recommended for the strawberry commercial quality. However, it failed in countering molds and yeast, the main spoilage microorganisms of strawberries; therefore, the addition of antimicrobial compounds was necessary [88].

Effectively, an emerging trend is the development of active packaging using functionalizing pectin films with antimicrobial compounds such as essential oils [105], polyphenols [90], nanomaterials [103], or free fatty acids [106], thus pointing out innovative strategies to create packaging with preservative-added properties. Xiong et al. [90] developed an edible coating for the preservation of a pork loin by loading a nanoemulsion composed of oregano essential oil and resveratrol into a pectin biopolymer matrix derived from a citrus peel. Meat is commonly preserved in high-oxygen modified atmosphere packaging (HOMAP), which has the shortcoming of an increase in the meat oxidation and leads to discoloration. The pectin coating on pork loins was effective in extending the shelf-life, by delaying protein and lipid oxidation, reducing pH change, maintaining meat tenderness, and showing an antimicrobial activity within 20 days of HOMAP packaging at 4 °C. The advantage deriving from the presence of oregano essential oils and resveratrol in the coating was not only to boost the preservative effect, due to their antimicrobial and antioxidant properties, but it also had a positive effect on tenderness and meat color [90]. Another example was the functionalization of citrus pomace pectin films with the rambutan (*Nephelium lappaceum*) peel extract (RPE). RPE incorporation increased the stretching ability and the light-blocking capacity of the film, making it suitable biodegradable film that could delay food lipid oxidation [91].

An eco-friendly intelligent food packaging material was recently developed by incorporating raspberry, blueberry, and blackberry waste extracts into pectin, exploiting the presence of anthocyanins as pH indicators for controlling the quality and freshness of fresh salmon. Blueberry extract pectin achieved the best results for film application, due to its higher mechanical properties, film density, pH changing color, and antioxidant capacities. This developed eco-friendly film was biodegradable in soil and seawater and succeeded in increasing the salmon shelf-life, highlighting an innovative food packaging material that could meet the market demands [89].

### 3.3. Emulsifying and Stabilizing Agent 

As previously mentioned, pectin is traditionally used as an emulsifying, gelling, stabilizing, and thickening agent to impart viscosity to different food and beverage products [103]. Its gelling performance is related to DM, which directly influences the gelation mechanism [107]. LMP can form cross-linking gels with divalent cations; differently, HMP requires a low pH (<3.5) and high amount of sugar (>65%) to gel, as it needs an acidic environment to reduce electrostatic repulsions between chains, and the presence of sugars close them together, with hydrogen bond formation [108]. Therefore, HMP is generally used as a food additive to produce commercial jam and jellies, which generally contain pectin in a range between 0.5 and 1.5% *w*/*w* [109]. However, the demand of low-calorie products by conscious consumers or by metabolic disorder patients has increased attention on LMP, which needs low sugar content to form gel due to the presence of low methoxy groups. A low-calorie jam for the glycemic control of type 2 diabetes patients was successfully formulated using low amidated citrus pectin with characteristics like those of commercial jam [110]. 

Moreover, pectin can be used as a food polysaccharide emulsifier to prepare food-grade emulsion in fruit drinks or milk products, thanks to the extent of hydrophobic methoxy and acetyl groups on its chains, able to reduce interfacial tension between the oil and water phase [111]. In addition, the presence of proteins covalently bound to side chains, neutral sugars, and ferulic acid, and the presence of acetyl groups, also influence the balance between hydrophilic and lipophilic regions, and thus the emulsifying properties of the polymer. Recently, sugar beet pectin was the most studied for its high creaming properties, thanks to the presence of proteins on polymer chains able to absorb oil droplets, decreasing surface tension with water [112]. Nevertheless, watermelon rinds gained attention as a new source of high-branching-degree pectin, with a small protein content—structural features that advise possible application as an emulsifying agent [58]. Despite the promising high DM, apple pectin did not present high emulsion capacity. Commercial citrus pectin had the lowest DM, but high HG content and flexibility, which allowed to better decrease surface tension in emulsions. However, pectin short chains were not able to give the steric hindrance required to avoid coalescence. In contrast, the combination of steric repulsion induced by the RG-I long chain of watermelon pectin and the protein content (which acts as surface materials) was able to generate stable O/W emulsions [60], pointing out a sustainable emulsifying pectin alternative to sugar beet pectin. Moreover, pectin could act as a stabilizing agent in milk products [113]. LMPs are mostly suitable as stabilizers in the production of low-fat yogurt, as casein calcium ions are sensitive to LM chains, promoting the penetration of hydrated pectin into the protein network. Thus, pectin carboxyl groups are able to absorb casein aggregates, leading to a stable homogeneous texture [109].

### 3.4. Functional Ingredient

Today, several biological functions are attributed to pectins depending on their conformation and degree of the branching chain [5], such as immunomodulatory [114], antioxidant, antiglycative [115], anti-inflammatory [94], cholesterol-lowering [116], and anti-cancer activity [117] that make them good candidates for the development of functional foods. According to Article 13 (1) of Regulation (EC) No. 1924/2006, EFSA approved different health claims related to pectin, such as the reduction in post-prandial glycemic responses (ID 786), the maintenance of normal blood cholesterol concentrations (ID 818), and the increase in satiety leading to a reduction in energy intake (ID 4692), for the relevance of the claimed effect on human health [11]. Pectin-derived oligosaccharides (POS) recently aroused attention as a new generation of prebiotics, as they stimulated the growth of colon bacteria with health benefits and could be obtained from pectin-rich food byproducts, meeting the principle of a circular economy [8]. POS exerted their activity by reaching, indigested, the colon and being selectively fermented by intestinal microbiota, with the production of short chain fatty acids (SCFA) (i.e., butyrate and acetate) that are selective substrates for lactobacilli and bifidobacteria growth, with efficiency similar to that of common commercial fructo-oligosaccharides (FOS) and galacto-oligosaccharides (GOS) [118,119]. POS are generally obtained from pectin depolymerization with enzymatic hydrolysis or other physicochemical approaches [118] and the subsequent fractionation of the degradation products [119]. DE, sugar composition, and MW were the main characteristics that affected pectin oligomers’ bioactivity on specific bacteria. Generally, HMP was more effective in producing a large amount of propionic acid in batch fecal fermentations rather than LMP [120]. Zhu et al. [115] succeeded in gaining more insight on POS structure–relation activity, characterizing pectin oligosaccharides obtained using enzymatic and UAE extraction from *Actidia arguta*. POS prebiotic activity was positively linked to the arabinose/rhamnose and galactose/rhamnose molar ratio and the presence of galactose and arabinose in molecule composition. However, POS oligosaccharides’ prebiotic activity on human microbiota is also related to the host age, as confirmed by a study of Wilkowska et al. [93], who evaluated the influence of childhood and elderly microbiota on the prebiotic effect of apple pomace POS, by using in vitro fecal fermentation. Microbiota in a 7-year-old child mainly formed bylactic acid bacteria, leading to higher SCFA and lactic acid production than in elderly subjects. Based on this effect, POS were effective in alleviating human dysbiosis disease, such as inflammatory bowel disease (IBD) [94] or obesity [121]. 

Concerning the anti-inflammatory effect of pectin, this activity seemed to be related to the neutral sugars present inside the chain. In fact, the removal of galactose from artichoke industrial byproduct pectin resulted in a loss of pectin bioactivity, consisting of reducing the expression of pro-inflammatory cytokines (TNF-a; IL-1b; IL-6) [94]. 

## 4. Conclusions

Although commercial pectin is primarily derived from citrus peels, apple pomace, and sugar beets, there has been a growing interest in utilizing alternative waste sources like tomato pomace, watermelon rinds, mangoes, bananas, melons, and eggplant peels to promote circular economy practices. These alternatives possessed comparable or even higher extraction yields than those obtained from citrus fruits.

The pectin chemical structure is strictly related to both its origin and the specific extraction method applied. The insights gathered from the literature examined in this manuscript indicate the most crucial factor determining the type of pectin extracted is the source of the waste material. Notably, the use of modern green extraction methods, such as MAE and UAE, can help provide a higher pectin yield in a short time compared to traditional approaches, facilitating the exploration of novel sources for industrial applications. As a result, a systematic optimization of the extraction process is mandatory, followed by a comprehensive characterization of the polysaccharide obtained in terms of DM, GalA content, MW distribution, and even techno-mechanical properties. By exploring these aspects thoroughly, we can better understand the potential commercial viability of pectin for various applications.

Pectin is traditionally used as a food additive to impart viscosity in jams and dairy products, but more recently LM pectin derived from citrus peel fruits has been found to be suitable for the formulation of low-calorie products, due to the low sugar content needed for gelation [51,110]. Moreover, several health claims have been assigned to pectin, supporting its use in functional food and food supplements. In this contest, POS derived from emerging food waste, such as from apple pomace [93] or artichoke byproducts [94], are gaining attention as a new generation of prebiotics with efficiency similar to that of the well-known FOS and GOS. Recently, pectin has also been reported to be an effective encapsulating agent for colon targeting in solid oral formulation, as it is resistant to an acidic gastric environment, preserving the release until the small intestine. By functionalizing pectin with other polymers (such as chitosan or oligochitosan) or proteins, formulations are stabilized and able to reach the colon undamaged, and therefore are suitable to deliver nutraceuticals in this district. Promising solutions have been found with pectins derived from watermelon rinds [100], yuzu [83], and citrus peels [84]. In addition, pectin meets the requirement as a new biodegradable and natural polymer in food packaging. Recently, citrus fruit waste pectin functionalization with polyphenols, essential oils, or antioxidant extracts provides antimicrobial or UV-light blocking capacities to food packaging, providing new smart packaging solutions [90,91,103]. 

The complexity of the pectin structure extracted from alternative food waste raw materials required further studies to better use them in the creation of value-added ingredients. In addition, in order to find innovative sources of food-grade pectin, standardized guidelines for their chemical characterization could be useful to compare different studies’ results and estimate their commercial application. 

## Figures and Tables

**Table 1:** Extraction method parameters and yields obtained from different food-waste-derived pectins.

Waste	Extraction Technique	Extraction Parameters	Yield	Reference
Conventional sources				
Mandarin citrus peel	CE	HNO_3_ pH 1.6; LSR 36:1 (*v*/*w*); 100 min	25.6%	[18]
Orange peel	s-MAE	pH 1.2; LSR 21.5:1 (*v*/*w*); 7 min; 400 W; surfactant Tween 80 8 g L^−1^	32.8 ± 0.8%	[19]
	CE	HCl 0.1 M pH 1; LSR 30:1 (*v*/*w*); 180 min; 90 °C	27.5 ± 0.6%	
Grapefruit peel	CE	H_2_SO_4_ 0.1 M; LSR 30:1 (*v*/*w*); 60 min; 80 °C	25.53%	[20]
	CE	Citric acid 0.1 M; LSR 30:1 (*v*/*w*); 60 min; 80 °C	24.54%	
Lemon/lime peel	MAE	Citric acid pH 1.5; 700 W; 3 min	25.31 ± 1.24%	[21]
Apple pomace	CE	Citric acid 2N pH 1.9; LSR 50:1 (*v*/*w*); 1 h	22%	[22]
	SWE	-	14.89%	[23]
	RFAE	Citric acid pH 2.2; 19 min; LSR 25:1 (*v*/*w*); 88 °C	11.24 ± 0.69%	[24]
Emerging sources				
Mango peel	CE	Citric acid 1.5%; LSR 40:1 (*v*/*w*); 21–80 °C	24.2–31.7%	[25]
Banana peel	CE	HCL 0.5 N pH 2.5; 2.5 h; LSR 10:1 (*v*/*w*); 90 °C	20–24%	[26]
Jackfruit peel	CE	Citric acid pH 2.0; LSR 17.03:1 (*v*/*w*); 9.15 min; 138 °C	16.83%	[27]
Watermelon rind	CE	Citric acid pH 2.0; LSR 35.07:1 (*v*/*w*); 62.31 min, 80 °C	24.30%	[28]
Pumpkin peel	CE	Citric acid pH 2.85; LSR 1:20 (*v*/*w*); 13 min; 89.98 °C	69.89 ± 2.90%	[29]
Melon peel	MAE	LSR 20.94:1 (*v*/*w*); 414.4 W; 12.75 min	32.81%	[30]
Eggplant peel	UAE	pH 1.5; 50 W; LSR 1:20 (*v*/*w*); 30 min	33.64%	[31]
Tomato waste	UAE	Citric acid pH 1.0; LSR 1:20 (*v*/*w*); 20 min; 80 °C	32.77%	[32]
Pistachio green hull	CE	Citric acid pH 0.5; LSR 1:50 (*v*/*w*); 30 min; 90 °C	22.1 ± 0.5%	[33]
Almond hull	CE	pH 1.4; LSR 20.13 (*v*/*w*); 58.65 min; 90 °C	26.32%	[34]

CE—conventional extraction; s-MAE—surfactant-microwave-assisted extraction; MAE—microwave-assisted extraction; SWE—subcritical water extraction; RFAE—radiofrequency-assisted extraction; UAE—ultrasound-assisted extraction; LSR—liquid–solid ratio.

**Table 2:** General overview of the recent applications of pectin in food and nutraceutical field.

Application	Goal	Type	Pectin Composition or Functionalization	Source	Ref.
Drug delivery	Colon targeting	Hydrogel beads	De-esterified HMP + oligochitosan cross-linked with Ca^2+^	Yuzu (*Citrus junus*) peel	[83]
		Biopolymer conjugated nanoliposome	HMP + chitosan	Citrus peel	[84]
	Controlled release	Aerogels	De-esterified HMP	Watermelon rind	[85]
	Co-encapsulation lipophilic–hydrophilic actives	Nanoparticles	LMP + zein	Citrus peel	[86]
Food packaging	Maintain quality and freshness	Edible films	HMP + cross-linked with Ca^2+^	Fig stalk	[87]
			HMP + glycerol	*Hibiscus Sabdariffa* byproducts	[88]
		pH–color changing coating	LMP + berries’ extract	Apple pomace	[89]
	Preservative properties	Packaging material	HMP + resveratrol and oregano essential oil	Citrus peel	[90]
	Inhibition of UV light	Packaging material	HMP + rambutan (*Nephelium* Lappaceum) peel extract	*Citrus junos* pomace	[91]
Food industry	Emulsifying agent	Food additive	Highly brunched HMP with small protein content	Watermelon rind	[60]
	Stabilizing agent	Food additive in yogurt	-	Banana and papaya peel	[92]
	Gelling agent	Food additive in jams	High-molecular-weight pectin	Jackfruit peel	[51]
Functional ingredient	Prebiotic activity	Pectin oligosaccharides	Range degree of polymerization 2–17	Apple pomace	[93]
	Anti-inflammatory activity	Pectin polysaccharide	Pectin with galactose units	Artichoke byproducts	[94]

HMP—high methoxyl pectin; LMP—low methoxyl pectin.

## Data Availability

The datasets used and/or analyzed during this study are available from the corresponding author upon reasonable request.
